# Considerations for the treatment of frail multiple myeloma patients

**DOI:** 10.46989/001c.92586

**Published:** 2024-01-31

**Authors:** Mohamad Mohty, S Vincent Rajkumar

**Affiliations:** 1 Sorbonne University, Service d’Hématologie Clinique Et de Thérapie Cellulaire, Hôpital Saint Antoine, AP-HP, INSERM UMRs938, Paris, France; 2 Division of Hematology, Mayo Clinic, 200 First Street, SW, Rochester, MN 55905, USA

**Keywords:** Multiple myeloma, Frailty, Therapy

The optimal treatment of frail patients with myeloma is not easily derived from available clinical trials, including ones that purportedly included such patients. For example, the MAIA trial^1^ included 737 multiple myeloma (MM) patients, of whom many were considered as “frail”. However, the data from the MAIA trial cannot be simply extrapolated to real-world patients, because many frail patients seen in daily and routine practice are not be the type of patients eligible for the MAIA trial (or other similar clinical trials). In fact, only a small subset, even among the group of elderly MM patients, might have been eligible. Therefore, we cannot generalize regimens and outcomes without knowing what sort of patients were studied in many of the MM trials that included elderly and/or frail patients. Even when trials have tried to include frail patients, inclusion and exclusion criteria permit enrolment of only patients with relatively good performance status, and most frail and very frail MM patients who need therapy are excluded. In the absence of good data, the treatment of frail patients typically relies on a physician’s “clinical judgement” in the selection of the regimen, and adjustment of dose and schedule. Importantly, if regimens used in clinical trials were generalized to truly frail patients without such adjustments, we could cause significant harm.

## Prognostic factors for survival in MM

In a large series published by Kyle et al. in 2003,^2^ of all the prognostic factors, the most powerful factor with an almost doubling in the relative risk (RR) was *performance status 3 or 4* (RR: 1.9, 95% CI 1.6-2.4; *p*<0.001). With performance status, a score of ≥2 is frail according to the simplified frailty system, but 2 is very different to 3 or 4. A score of 3 means that the patient is not up and about for more than half the time they are awake. Performance status 3 or 4 is not represented in almost all clinical trials in MM, and such patients have a poorer prognosis, even compared to patients with biologic high-risk disease.

In the MAIA trial, almost half of the patients included were considered to be frail, but this was primarily driven by age >80 years. In fact, 16% of the patients had a performance status ≥2, but the majority of this group had a score of 2, and not 3 or 4. Only 1.3% of patients in this trial had a creatinine clearance of <30ml/min, and so the really frail patients with advanced renal failure were not included.

In a recent systematic review^3^ by Mian et al., the prevalence of frailty in MM ranges from 17% to 70%. We also need to acknowledge that frailty is a dynamic concept, and this area has largely been unexplored, so a one-time frailty assessment is far from sufficient.

## Recommended Approach to Initial Therapy of frail MM patients

The most common two regimens used world-wide in the treatment of NDMM are VRd (bortezomib, lenalidomide, and dexamethasone) and DRd (daratumumab in combination with lenalidomide and dexamethasone). With such regimens, patients will survive for longer than 4 or 5 years, provided they are able to receive these regimens at the right dose and schedule. In the updated analysis of the SWOG S0777 trial, the median overall survival (OS) with VRd was >65 months, and the 5-year OS was 55%. In the MAIA trial, the median OS with DRd was >60 months, with a 5-year OS of 66%. Both these regimens are good options for newly diagnosed myeloma in patients who are not candidates for stem cell transplantation. However, at present, we do not have data from randomized clinical trials comparing VRd or DRd, and very limited data to guide therapy for frail patients. These two trials cannot be easily compared since the inclusion criteria varied significantly. The SWOG trial^4^ included only 39% of patients aged >65 years, whereas almost all (99%) patients in the MAIA trial were in this age group. Thus, one must make judgement calls when selecting between these two regimens. One consideration is that with VRd, we give three drugs only for six months, followed by oral lenalidomide or lenalidomide plus dexamethasone. With DRd, however, the daratumumab-based triplet is continued monthly until progression, for perhaps 5 or 6 years.

Some considerations for choice of initial therapy relates to the baseline status of the patient; neuropathy risk *versus* infection risk when deciding on a regimen. Cost is another important key parameter. Bortezomib is now inexpensive in most parts of the world whereas daratumumab remains very expensive. Thus, access and cost must be considered as part of the decision-making process. Convenience and feasibility are also important as very frail or elderly patients may not be able to visit monthly for daratumumab and may be only able to get oral therapy.

In patients who are considered too frail to be able to tolerate triplet therapy, one could use just the Rd doublet as shown in the FIRST trial,^5^ and consider the addition of daratumumab later in the disease course (eg. first relapse). In patients with acute renal failure for whom up-front lenalidomide is not feasible, we usually rely on regimens such as daratumumab, bortezomib, dexamethasone (DVd) or bortezomib, cyclophosphamide, dexamethasone (VCd).

A key factor besides selecting the appropriate regimen for initial therapy is selecting the optimal dose and duration. There is a true gap between the recommended or approved dosage of a given drug, and the actual dose a patient can receive. For example, if VRd is used as per the SWOG^4^ trial (twice weekly, intravenous), many patients will experience a high rate of neuropathy compared to the preferred once-weekly subcutaneous dosing. In frail patients it becomes even more important to adjust the dose and schedule according the patients’ clinical and functional status and comorbidities. This was the concept behind the “VRd-lite” regimen, where both the lenalidomide and dexamethasone doses are reduced, while bortezomib is given once per week subcutaneously.^6^ This regimen has proved to be useful in frail patients. The same can be applied to the DRd regimen, by just reducing the dose of lenalidomide to 10 or 15 mg. The dexamethasone dose is commonly reduced to 20 mg once per week in patients age>70, but for elderly frail patients this can be further reduced if necessary. In fact, we recommend stopping dexamethasone after a few months in responding frail patients, so as to reduce the risk of infections and serious complications in the long-term. The ultimate goal should be to adjust the dose, and duration of therapy to achieve a situation whereby the patient can tolerate the treatment and receive it for long enough.

## Future perspectives for frail MM patients

For the clinical trial-eligible frail patient (e.g. a frail patient aged >80 years who has no heart failure, and does not have performance status 3 or 4), the biologic MM risk has to be considered as a priority because often one needs to administer treatments differently, particularly for maintenance. On the other hand, for very frail patients (performance status 3 or 4, or multiple comorbidities), we need to be careful in selecting the appropriate regimen, dose and schedule, and be willing to initiate therapy with doublet or even monotherapy until the clinical condition allows escalation if needed. These patients will have to be studied in clinical trials based on frailty scores. An ongoing trial by ECOG (Eastern Cooperative Oncology Group) of patients who might not have qualified for the MAIA trial, is investigating VRd-lite *versus* DRd-lite. The use of the simplified frailty index based on performance status, comorbidity index, and age, should be encouraged in routine clinical practice. The use of this score will help managing elderly patients not eligible to clinical trial regimens, especially if they cannot receive a triplet regimen.^7^

### ETHICAL APPROVAL

Not applicable.

### CONSENT TO PARTICIPATE/INFORMED CONSENT

Not applicable.

### CONSENT FOR PUBLICATION

Not applicable.

### COMPETING INTERESTS/CONFLICT OF INTEREST

Mohamad Mohty received lectures and consultancy honoraria from Adaptive Biotechnologies, Amgen, Astellas, BMS, GSK, Janssen, Jazz Pharmaceuticals, Novartis, Pfizer, Sanofi, Stemline Therapeutics, and Takeda, all outside the scope of this manuscript.

### AUTHORS’ CONTRIBUTION – CREDIT TAXONOMY

Conceptualization: Mohamad Mohty (Equal), S Vincent Rajkumar (Equal). Writing – original draft: Mohamad Mohty (Lead). Writing – review & editing: Mohamad Mohty (Equal), S Vincent Rajkumar (Equal).
